# The Good Still Increases

**Published:** 1897-08

**Authors:** 


### THE GOOD STILL INCREASES.

THE failure of Governor Pingree, of Michigan, to reappoint as State Veterinarian Dr. E. A. A. Grange, who so well filled the position for so many years, will not be without its compensation to the profession, who will rejoice in the knowledge that he has been called by the Detroit Veterinary College to take charge of that institution, and with the power granted and instruction given to make this school one of the best in the country, in full accord with all the leading veterinary institutions of our land. It will at once be made a three-years' curriculum of six months each, and the warm place Dr. Grange has always held among his colleagues will enable him to call to his aid the best men of that section of our country.

The authorities of the Detroit College are to be congratulated on so wise a selection. They secure in Dr. Grange a well-equipped man by experience, training, and ability, and we are very sure that this school will now take a prominent place in the galaxy of schools and add a powerful influence to the movement for higher veterinary education in America. It is unfortunate that the State loses so valuable an officer and the Agricultural College so efficient a teacher, but the wider sphere of his usefulness in his new field will bring to the whole State a greater return than can be measured at this time.

